# Dynamic ferritin levels and survival outcomes in secondary hemophagocytic syndrome: a comprehensive analysis

**DOI:** 10.3389/fmed.2025.1517101

**Published:** 2025-05-15

**Authors:** Gorkem Koymen, Sukriye Miray Kilincer Bozgul, Ilkce Akgun Kurtulmus, Nur Akad Soyer, Gunes Ak, Devrim Bozkurt, Ajda Gunes, Figen Yargucu Zihni

**Affiliations:** ^1^Division of Medical Oncology, Department of Internal Medicine, Izmir City Hospital, Izmir, Türkiye; ^2^Division of Intensive Care, Department of Internal Medicine, Faculty of Medicine, Ege University, Izmir, Türkiye; ^3^Clinic of Internal Medicine, Torbalı State Hospital, Izmir, Türkiye; ^4^Division of Hematology, Department of Internal Medicine, Faculty of Medicine, Ege University, Izmir, Türkiye; ^5^Department of Clinical Biochemistry, Faculty of Medicine, Ege University, Izmir, Türkiye; ^6^Division of Rheumatology, Department of Internal Medicine, Faculty of Medicine, Ege University, Izmir, Türkiye

**Keywords:** hemophagocytic syndrome, ferritin, prognostic markers, survival analysis, treatment outcomes

## Abstract

**Background:**

Hemophagocytic syndrome (HPS) is a life-threatening condition characterized by excessive immune activation and cytokine release. Reliable prognostic markers to guide clinical management and predict outcomes are lacking. This study aims to evaluate the prognostic ability of dynamic ferritin levels in patients with HPS and their potential role in guiding treatment modifications.

**Materials and methods:**

We conducted a retrospective cohort study including 99 patients diagnosed with HPS between January 2013 and June 2022. Data on demographic characteristics, clinical presentations, treatment modalities, and laboratory values were collected, including ferritin levels at diagnosis and during treatment. Statistical analyses including Cox proportional hazards models were used to assess the risk factors of mortality, and a receiver operating curve analysis was conducted to assess the association between ferritin level changes and survival outcomes.

**Results:**

Patients exhibiting a decrease in ferritin levels within the first week of treatment had significantly improved survival rates compared to those with increasing levels (*p* < 0.05). Multivariate analysis identified early ferritin level reduction as an independent predictor of better survival outcomes (HR 0.62, 95% CI: 0.41–0.93, *p* = 0.02). Notably, decreases below 7.54% in ferritin levels from day 0 to day 3 were associated with 85.19% sensitivity and 57.14% specificity for survival prediction (AUC = 0.684), while increases >36.67% from day 0 to day 5 showed 50.0% sensitivity and 85.0% specificity for mortality prediction (AUC = 0.697). Additionally, the liver injury group demonstrated significantly higher ferritin levels and D-dimer values, with lower fibrinogen and albumin levels compared to the non-liver injury group.

**Conclusion:**

The improved survival associated with early decreases in ferritin levels highlights the potential of ferritin monitoring to guide clinical decision-making and treatment adjustments. Based on the results, the integration of ferritin level dynamics into HPS management protocols could improve patient outcomes.

## 1 Introduction

Hemophagocytic syndrome (HPS), otherwise known as hemophagocytic lymphohistiocytosis (HLH), is a life-threatening clinical emergency condition that develops as a result of over-activation of the immune response and loss of self-limitation in inflammatory processes. Primary (familial) HLH is usually driven by genetic abnormalities in childhood, while secondary forms are triggered by environmental and acquired mechanisms, such as autoimmunity, malignancy, and infections (1). The underlying pathogenesis of HPS, regardless of whether it is primary or secondary, involves the development of defects in the function of natural killer (NK) cells and cytotoxic T cells, resulting in excessive immune activation and cytokine storm, leading to multi-organ failure (2, 3). Organ failure in HPS patients frequently necessitates admission to the intensive care unit (ICU). Clinical symptoms include fever, cytopenias, hepatitis, coagulopathy, heart failure, and other manifestations. Mortality rates between 40% and 80% have been reported in HPS patients (4–6). The criteria used to diagnosis HPS were last updated in 2004 (7). It is important to note that the HLH-2004 diagnostic criteria were originally developed for pediatric patients with primary HLH and have not been formally validated in adult populations. Additionally, Fardet et al. (8) developed the H-score to estimate the risk of secondary HPS. Clinical and demographic factors along with laboratory examinations, which are commonly used in daily practice, have been investigated in many studies as prognostic factors (9–11). However, the current parameters are non-specific and make predicting the prognosis of HPS particularly challenging. Among laboratory findings, one parameter that is involved both in the pathogenesis and prognosis of HPS is ferritin (12), and thus it may be useful in determining the patient's prognosis (13). Rather than a single measurement, dynamic changes, especially declining ferritin levels, have been proposed as prognostic markers in critically ill patients (4, 5, 14). Mortality during the first week of HPS is high due to multi-organ failure (15), and there is a need for parameters to guide decision-making and treatment intensity during this critical period. However, there is a lack of information on how and when declining ferritin levels can serve as a guide for monitoring patients' treatment response. Accordingly, in this retrospective cohort study, we aimed to determine the prognostic role of dynamic ferritin levels throughout the treatment process and ICU survival outcomes of HPS patients.

## 2 Materials and methods

### 2.1 Study participants and data acquisition

This retrospective cohort study was conducted in the internal medicine ICU of Ege University Hospital between January 2013 and June 2022. The study was approved by the Ege University Ethics Committee (22-7T/66) and adhered to the principles of the Declaration of Helsinki. HPS patients meeting ≥ five of the eight HLH-2004 (7) diagnostic criteria and having serial ferritin measurements were included in the study. Patients who were younger than 18 years of age, had a history of terminal stage malignancy, died within the first 24 h, and had missing data were excluded from the study. Patients or their relatives provided written informed consent. Data on demographic characteristics, clinical presentations, treatment modalities, and laboratory values, including ferritin levels at diagnosis (which was also the first day of treatment) and during follow-up, were collected. H-scores were calculated following Fardet et al. (8). The underlying causes for the etiology of HPS were evaluated by a team including a hematologist, a rheumatologist, and an experienced intensive care physician.

### 2.2 Statistical analysis

The data were summarized using descriptive statistics. For continuous (numerical) variables, depending on the distribution, either the mean ± standard deviation or median, minimum, and maximum values were presented in a tabular form. Categorical variables were summarized as counts and percentages. The normality of numerical variables was assessed using Shapiro-Wilk, Kolmogorov-Smirnov, and Anderson-Darling tests. To compare differences in categorical variables between groups, the Pearson chi-square test was used for 2 × 2 tables with five or more expected observations, while Fisher's exact test was employed for tables with five or fewer expected observations. The Fisher Freeman Halton test was utilized for R × C tables with less than five expected observations. In comparisons between two independent groups, the independent samples *t*-Test was used when the numerical variables demonstrated normal distribution, while the Mann-Whitney *U*-test was applied in cases of non-normal distribution. Cox's proportional hazard model was utilized to identify factors affecting the duration of hospitalization and mortality in patients with HPS, based on both univariate and multivariate Cox regression analyses. In the univariate Cox regression analyses, the impact of each independent variable, including age, H-score at diagnosis, albumin, lactate dehydrogenase (LDH), platelet count, and the percentage change in ferritin between admission day (D0)–third day of treatment (D3) and D0–fifth day of treatment (D5), on mortality was examined separately. Hazard ratios (HRs), 95% confidence intervals (CIs), and *p-values* were calculated for each variable. In the multivariate Cox regression analyses, since the combined effects of variables other than the Δ% ferritin (D0–D3) change were not significant, only the impact of this variable on the risk of mortality was assessed, and HRs, 95% CIs, and *p-values* were presented. A receiver operating characteristic (ROC) analysis was conducted to evaluate the effectiveness of Δ% ferritin changes (D0–D3 and D0–D5) in predicting mortality. The analysis measures the ability of Δ% ferritin change rates to distinguish mortality outcomes, including area under the curve (AUC), sensitivity, specificity, and optimal cut-off values. For both time intervals, AUC, sensitivity, specificity, cut-off values, and 95% CIs were calculated and presented. AUC values and *p-values* indicated the statistical significance of the capability of Δ% ferritin change rates to predict mortality. Cut-off values for percentage changes in ferritin levels (Δ% ferritin) were determined using receiver operating characteristic (ROC) curve analysis. Optimal cut-off points were selected as values providing the best combination of sensitivity and specificity for predicting mortality, based on the principle of Youden's index maximization (sensitivity + specificity – 1). Additionally, tertiles were evaluated according to data distribution, and similar studies in the literature were considered to enhance clinical applicability.

In addition to predefined variables, we retrospectively calculated the ferritin-to-platelet ratio at admission by dividing the serum ferritin level (ng/mL) by the platelet count (×103/μL), in order to explore its prognostic relevance.

Statistical analyses were conducted using Jamovi (Version 2.3.28) and JASP (Version 0.17.3) software, and the level of significance for the statistical analyses was set at 0.05 (*p-value*).

## 3 Results

This study included 99 patients diagnosed with HPS, with an average age of 48.8 ± 16.7 years. Of the patients, 46 (46.5%) were male, and 53 (53.5%) were female. The median duration of hospitalization was 12 days. Etiologically, HPS was linked to infections in 32 patients (32.3%), rheumatological conditions in 34 patients (34.3%), malignancies in 17 patients (17.2%), and other causes in 16 patients (16.2%). Treatment modalities included cyclosporine for 15 patients (15.2%), intravenous immunoglobulin (IVIG) for 88 patients (88.9%), and methylprednisolone for 82 patients (82.8%). A combination of IVIG and methylprednisolone was administered to 71 patients (71.7%), cyclophosphamide to 23 patients (23.2%), and plasmapheresis to 16 patients (16.2%). Bone marrow aspiration and biopsy were performed in 82 patients (82.8%). The average initial H-score was 190.8 ± 50.0, increasing to a maximum average of 219.0 ± 52.5 during follow-up.

Age, initial H-scores, and maximum H-scores were significantly higher at follow-up for patients who succumbed to HPS (*p* < 0.05 for all). Plasmapheresis was significantly more common in patients who died (*p* = 0.047). No significant differences were observed between survivors and non-survivors in terms of gender, hospital stay duration, etiological factors, and treatments, including cyclosporine, IVIG, methylprednisolone, cyclophosphamide, and the combination of IVIG and methylprednisolone, with *p-values* >0.05 for each (Table 1).

**Table 1:** Descriptive statistics and pairwise comparisons of demographic and clinical features in patients with hemophagocytic syndrome.

**Variables**	**Overall (*n* = 99)**	**Mortality**		***p*-values**
		**Survived (*****n*** = **62)**	**Deceased (*****n*** = **37)**	
Age	48.8 ± 16.7	44.0 ± 15.5	56.7 ± 15.8	**<0.001**
**Gender**				
Male	46 (46.5)	29 (46.8)	17 (45.9)	0.999
Female	53 (53.5)	33 (53.2)	20 (54.1)	
Duration of hospital stay (Days)	12.0 (4.0 – 92.0)	15.0 (4.0 – 73.0)	11.0 (4.0 – 92.0)	0.343
Time from diagnosis to treatment (Days)	12.0 (1.0 – 82.0)	13.0 (1.0 – 56.0)	10.0 (1.0 – 82.0)	0.635
**Etiology**				
Related to infection	32 (32.3)	20 (32.3)	12 (32.4)	0.49
Related to rheumatologic disease	34 (34.3)	23 (37.1)	11 (29.7)	
Related to malignancy	17 (17.2)	8 (12.9)	9 (24.3)	
Other etiology	16 (16.2)	11 (17.7)	5 (13.5)	
Cyclosporine treatment, Yes	15 (15.2)	10 (16.1)	5 (13.5)	0.951
Intravenous immunoglobulin treatment, Yes	88 (88.9)	53 (85.5)	35 (94.6)	0.202
Methylprednisolone treatment, Yes	82 (82.8)	51 (82.3)	31 (83.8)	0.999
IVIG and methylprednisolone, Yes	71 (71.7)	43 (69.4)	28 (75.7)	0.656
Cyclophosphamide treatment, Yes	23 (23.2)	17 (27.4)	6 (16.2)	0.303
Plasmapheresis, Yes	16 (16.2)	6 (9.7)	10 (27.0)	**0.047**
Bone marrow aspiration and biopsy, Yes	82 (82.8)	50 (80.6)	32 (86.5)	0.638
H score at diagnosis	190.8 ± 50.0	183.0 ± 50.1	203.9 ± 47.7	**0.042**
Maximum H score during follow-up	219.0 ± 52.5	203.6 ± 50.7	244.9 ± 45.2	**<0.001**

IVIG, Intravenous immunoglobulin. *p* values that are statistically significant were written in bold.

Laboratory analysis revealed median values for various markers, with significant differences in mortality associated with the international normalized ratio (INR), triglycerides, direct bilirubin, and platelet levels. Specifically, platelet levels were significantly higher in surviving patients (*p* = 0.022, respectively), while INR, direct bilirubin, and triglyceride levels were significantly higher in deceased patients (*p* < 0.05 for each). No significant differences were found for fibrinogen, high-density lipoprotein cholesterol, albumin, urea, creatinine, alanine transaminase, aspartate aminotransferase, LDH, C-reactive protein (CRP), procalcitonine, hemoglobulin, neutrophils, lymphocytes, or the neutrophile-to-lymphocyte ratio (NLR) (*p* > 0.05 for each) (Table 2).

**Table 2:** Descriptive statistics of initial hematological and biochemical parameters in patients with hemophagocytic syndrome and pairwise comparisons for mortality.

**Variables**	**Overall (*n* = 99)**	**Mortality**		***p*-values**
		**Survived (*****n*** = **62)**	**Deceased (*****n*** = **37)**	
Fibrinogen (mg/dL)	333.5 (38.0 – 1,050.0)	346.0 (58.0 – 986.0)	304.5 (38.0 – 1,050.0)	0.251
INR	1.1 (0.8 – 3.3)	1.1 (0.8 – 2.6)	1.2 (0.9 – 3.3)	**0.019**
HDL-cholesterol (mg/dL)	13.0 (4.0 – 51.0)	15.0 (5.0 – 51.0)	12.0 (4.0 – 49.0)	0.147
Triglycerides (mg/dL)	230.0 (9.0 – 1,091.0)	208.0 (9.0 – 873.0)	277.5 (70.0 – 1,091.0)	**0.025**
Albumin (g/dL)	2.8 ± 0.6	2.9 ± 0.6	2.6 ± 0.6	0.071
Urea (mg/dL)	49.0 (12.0 – 278.0)	39.5 (12.0 – 277.0)	61.0 (21.0 – 278.0)	0.059
Creatinine (mg/dL)	0.9 (0.2 – 12.1)	0.8 (0.3 – 12.1)	1.2 (0.2 – 6.3)	0.286
ALT (U/L)	42.0 (5.0 – 2,134.0)	41.0 (5.0 – 1,380.0)	47.0 (5.0 – 2,134.0)	0.474
AST (U/L)	68.0 (6.0 – 7,435.0)	50.5 (6.0 – 1,115.0)	78.0 (6.0 – 7,435.0)	0.155
LDH (U/L)	527.0 (126.0 – 9,026.0)	514.0 (134.0 – 4,408.0)	769.0 (126.0 – 9,026.0)	0.086
Direct bilirubin (mg/dL)	0.6 (0.1 – 22.3)	0.5 (0.1 – 11.2)	0.9 (0.1 – 22.3)	**0.008**
CRP (mg/dL)	10.0 (0.1 – 99.1)	10.6 (0.1 – 37.6)	7.6 (0.4 – 99.1)	0.452
Procalcitonin (ng/mL)	1.3 (0.1 – 100.0)	1.1 (0.1 – 100.0)	1.6 (0.1 – 100.0)	0.763
Hemoglobin (g/dL)	9.3 (3.6 – 18.3)	9.1 (3.6 – 18.3)	9.6 (6.2 – 17.6)	0.184
Platelet (cells/μL)	76,000.0 (3,000.0 – 579,000.0)	82,000.0 (7,000.0 – 579,000.0)	62,000.0 (3,000.0 – 329,000.0)	**0.022**
Neutrophils (cells/μL)	2,870.0 (0.0 – 33,000.0)	2,725.0 (0.0 – 33,000.0)	3,450.0 (30.0 – 31,080.0)	0.707
Lymphocytes (cells/μL)	660.0 (0.0 – 6,150.0)	665.0 (0.0 – 3,940.0)	660.0 (80.0 – 6,150.0)	0.854
NLR	4.4 (0.0 – 82.5)	4.8 (0.0 – 68.7)	3.8 (0.1 – 82.5)	0.710
Ferritin D0 (ng/mL)	2,842.0 (500.0 – 169,766.0)	1,851.0 (511.0 – 73,500.0)	4,301.0 (500.0 – 169,766.0)	0.087
Ferritin-to-platelet ratio	0.1 (0.0 – 10.6)	0.0 (0.0 – 2.4)	0.1 (0.0 – 10.6)	0.011

INR, International normalized ratio; HDL, High-density lipoprotein; ALT, Alanine aminotransferase; AST, Aspartate aminotransferase; LDH, Lactate dehydrogenase; NLR, Neutrophil to lymphocyte ratio. *p* values that are statistically significant were written in bold.

**Figure 1:**
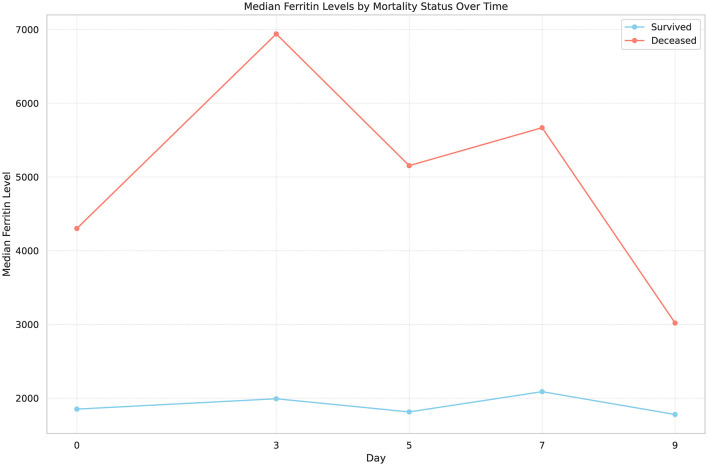
Dynamic trends of median ferritin levels across different days highlighting mortality outcomes in hemophagocytic syndrome patients.

The study also monitored changes in ferritin levels over time among patients with HPS. Median ferritin levels were measured as 2842.0 ng/mL on day 0, increasing to 3458.0 ng/mL by day 3, then decreasing to 2699.5 ng/mL by day 5, 2464.0 ng/mL by day 7, and finally to 1937.0 ng/mL by day 9 of treatment. When comparing ferritin levels on days 3, 5, and 7 of treatment in relation to mortality, the levels were significantly higher in patients who died than in survivors (*p* < 0.05 for each). However, ferritin levels on days 0 and 9 showed no significant difference based on mortality status (*p* = 0.087 and *p* = 0.294, respectively). Additionally, no significant difference was observed in the overall sample and between the surviving and deceased patient groups when analyzing the change in ferritin levels (*p* = 0.165, *p* = 0.248, *p* = 0.504, respectively) (Table 3). Patients who failed to show a decrease in ferritin levels during the first 5 days of treatment had significantly higher mortality, indicating the prognostic importance of early ferritin kinetics. Figure 1 shows the dynamic trends of median ferritin levels across different days.

**Table 3:** Within-group and between-group comparisons of ferritin levels in patients with hemophagocytic syndrome.

**Variables**	**Overall (*n* = 99)**	**Mortality**		***p*-values**
		**Survived (*****n*** = **62)**	**Deceased (*****n*** = **37)**	
Ferritin D0 (ng/mL)	2,842.0 (500.0 – 169,766.0)	1,851.0 (511.0 – 73,500.0)	4,301.0 (500.0 – 169,766.0)	0.087
Ferritin D3 (ng/mL)	3,458.0 (190.0 – 173,000.0)	1,991.5 (190.0 – 30,251.0)	6,940.0 (615.0 – 173,000.0)	**0.028**
Ferritin D5 (ng/mL)	2,699.5 (204.0 – 59,154.0)	1,813.0 (204.0 – 59,154.0)	5,153.5 (431.0 – 56,666.0)	**0.021**
Ferritin D7 (ng/mL)	2,464.0 (438.0 – 84,463.0)	2,087.5 (438.0 – 39,912.0)	5,668.0 (543.0 – 84,463.0)	**0.040**
Ferritin D9 (ng/mL)	1,937.0 (181.0 – 120,659.0)	1,778.0 (490.0 – 35,299.0)	3,019.0 (181.0 – 120,659.0)	0.294
*p*-values	0.165	0.248	0.504	

D, Day. *p* values that are statistically significant were written in bold.

The median length of hospital stay among non-survivors, used here as a proxy for time to death, was 11 days (range: 4–92 days). In addition, we retrospectively calculated the ferritin-to-platelet ratio on admission by dividing serum ferritin level (ng/mL) by platelet count (×103/μL). This ratio was significantly higher in non-survivors compared to survivors [0.1 (0.0–10.6) vs. 0.0 (0.0–2.4), *p* = 0.011]. The overall median ferritin-to-platelet ratio was 0.1 (0.0–10.6). These findings suggest that the ferritin-to-platelet ratio may serve as a practical and accessible prognostic marker in HPS.

Ferritin percentage changes (Δ%) were assessed on days 3, 5, 7, and 9. The median Δ% values for ferritin were −1.73% for days 0–3, −5.23% for days 0–5, −13.17% for days 0–7 days, and −32.34% for days 0–9. When comparing the changes in ferritin levels between the groups of patients who survived and those who exited during the follow-up period, it was found that in patients who survived during the follow-up, ferritin levels decreased compared to baseline on days 3 and 5, whereas in patients who died during the follow-up, ferritin levels increased. The difference between the two groups was statistically significant (*p* = 0.010 and *p* = 0.010, respectively). However, on days 7 and 9, there was no significant difference in ferritin level changes compared to baseline between the two groups (*p* = 0.157 and 0.660, respectively) (Table 4).

**Table 4:** Comparison of % change in ferritin levels between groups in patients with hemophagocytic syndrome.

**Variables**	**Overall (*n* = 99)**	**Mortality**		***p*-values**
		**Survived (*****n*** = **62)**	**Deceased (*****n*** = **37)**	
Ferritin Δ % (0–3)	−1.7 (−89.5 to 8,751.6)	−11.9 (−89.5 to 324.8)	7.6 (−71.4 to 8,751.6)	**0.010**
Ferritin Δ % (0–5)	−5.2 (−95.5 to 2,901.4)	−35.3 (−90.3 to 1,822.4)	28.1 (−95.5 to 2,901.4)	**0.010**
Ferritin Δ % (0–7)	−13.2 (−92.8 to 4,373.7)	−29.2 (−92.8 to 1,761.6)	6.2 (−91.6 to 4,373.7)	0.157
Ferritin Δ % (0–9)	−32.3 (−97.5 to 527.0)	−30.8 (−95.2 to 527.0)	−34.5 (−97.5 to 484.3)	0.660

Δ, percentage change in ferritin. *p* values that are statistically significant were written in bold.

According to the univariate Cox regression model, only the change in ferritin levels from day 0 to day 3 (Δ% ferritin D0–D3) significantly affected the risk of in-hospital mortality (*p* = 0.023), with each unit increase in Δ% ferritin on D0–D3 raising the mortality risk by 1%. Additionally, a significant effect of the changes in ferritin values on day 5 compared to baseline (Δ% ferritin D0–D5) was observed (*p* = 0.002). Therefore, a one-unit increase in ferritin percentage change increases the risk of mortality by 1%. Age, H-score at diagnosis, albumin levels, LDH levels, and platelet count did not significantly influence in-hospital mortality risk (*p* > 0.05 for each). The multivariate Cox regression model also highlighted the significance of the percentage change in ferritin levels from day 0 to day 5 (Δ% ferritin D0–D5) (*p* = 0.001), where each unit increase was associated with a 1% increase in the risk of inpatient mortality. However, no significant effect was observed regarding the percent change in day 3 ferritin levels (Δ% ferritin D0–D3) compared to baseline. Additionally, age was also found to have a significant effect (*p* = 0.017) (Table 5).

**Table 5:** Cox regression models for factors influencing mortality risk in patients with hemophagocytic syndrome.

**Cox's proportional hazard model on time (“Hospitalization Time”) to event (“Mortality”)**	**Univariate Cox regression**		**Multivariate Cox regression**	
	**HR (95%CI)**	***p*** **-values**	**HR (95%CI)**	***p*** **-values**
Age	1.03 (0.99 – 1.06)	0.070	1.02 (0.99 – 1.05)	**0.017**
H Score at Diagnosis	1.01 (0.99 – 1.01)	0.672	–	–
Albumin	0.78 (0.39 – 1.57)	0.486	–	–
LDH	1.01 (0.99 – 1.01)	0.997	–	–
Platelets	1.01 (1.00 – 1.01)	0.304	–	–
Δ% Ferritin (D0–D3)	1.01 (1.01 – 1.02)	**0.023**	1.01 (1.01 – 1.02)	**0.894**
Δ% Ferritin (D0–D5)	1.01 (1.01 – 1.02)	**0.002**	1.01 (1.01 – 1.02)	**0.001**

CI, Confidence interval; HR, Hazard ratio. *p* values that are statistically significant were written in bold.

**Figure 2:**
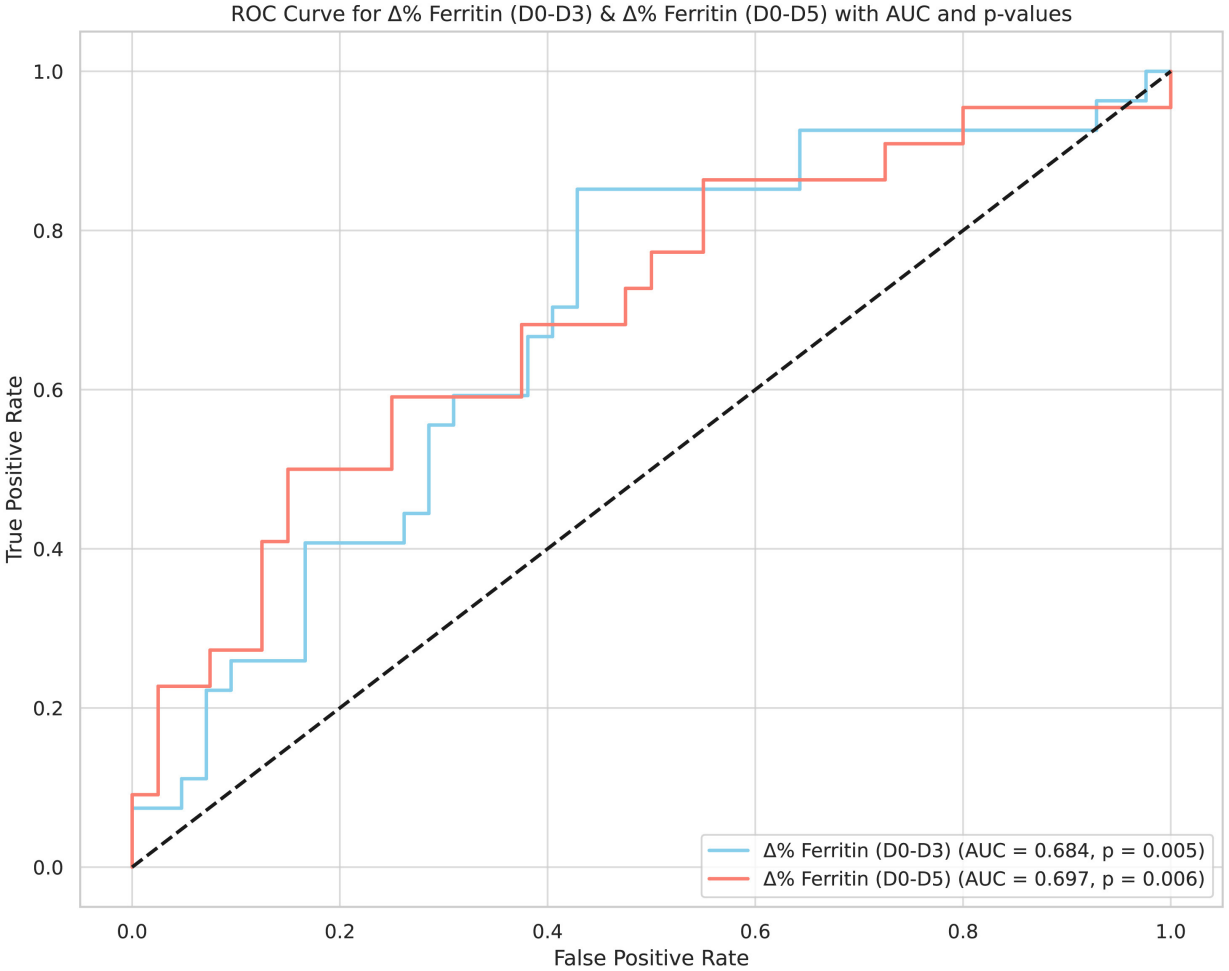
ROC analysis of Δ% ferritin changes for mortality prediction in hemophagocytic syndrome: a comparative study of early time points.

We also evaluated the role of the percentage change in ferritin levels from day 0 to day 3 (Δ% ferritin D0–D3) and from day 0 to day 5 (Δ% ferritin D0–D5) in predicting mortality. The results showed that decreases below the cut-off point of 7.54% for Δ% ferritin on D0–D3 had a sensitivity of 85.19%, a specificity of 57.14%, and an AUC of 0.684 (95% CI: 0.561–0.791, *p* = 0.005). Further, increases greater than the cut-off point of 36.67% for Δ% ferritin on D0–D5 had a sensitivity of 50.0%, a specificity of 85.0%, and an AUC of 0.697 (95% CI: 0.567–0.807, *p* = 0.005), respectively (Table 6; Figure 2).

**Table 6:** ROC analysis results for the diagnostic value of Δ% ferritin (D0–D3) and Δ% ferritin (D0–D5) values for mortality in patients with hemophagocytic syndrome.

**Variables**	**AUC**	**Sensitivity**	**Specificity**	**Cut off**	**95% CI**	***p*-values**
Δ% Ferritin (D0–D3)	0.684	85.19	57.14	>-7.54	0.561 to 0.791	**0.005**
Δ% Ferritin (D0–D5)	0.697	50.00	85.00	>36.67	0.567 to 0.807	**0.006**

CI, Confidence interval; AUC, Area under the curve. *p* values that are statistically significant were written in bold.

We also analyzed ferritin levels and their percentage changes according to the underlying etiology of HPS (Table 7). Although patients with malignancy-related and other etiology groups seemed to have higher baseline and follow-up ferritin levels compared to infection-related and rheumatologic disease-related groups, these differences did not reach statistical significance (*p* > 0.05 for all time points). Similarly, there were no significant differences in the percentage changes of ferritin levels among the four etiological groups at any time point (*p* > 0.05 for all comparisons). These findings suggest that the dynamics of ferritin levels in HPS patients follow similar patterns regardless of the underlying etiology.

**Table 7:** Ferritin levels and changes according to etiology in patients with hemophagocytic syndrome.

**Parameters**	**Infection-related (*n* = 32)**	**Rheumatologic disease-related (*n* = 34)**	**Malignancy-related (*n* = 17)**	**Other etiology (*n* = 16)**	***p*-values**
Ferritin D0 (ng/mL)	1,839.5 (539.0, 169,766.0)	2,073.0 (500.0, 26,732.0)	3,262.0 (511.0, 78,503.0)	5,097.5 (580.0, 84,692.0)	0.294
Ferritin D3 (ng/mL)	19,12.5 (190.0, 173,000.0)	1,946.0 (699.0, 44,258.0)	7,344.0 (583.0, 73,202.0)	6,625.0 (591.0, 92,688.0)	0.442
Ferritin Δ% (0–3)	−2.3 (−64.8, 324.8)	−10.8 (−71.4, 8,751.6)	−1.7 (−61.2, 870.5)	3.1 (−89.5, 80.1)	0.789
Ferritin D5 (ng/mL)	2,958.5 (259.0, 59,154.0)	2,535.0 (204.0, 27,757.0)	4,799.0 (547.0, 32,325.0)	3,100.0 (547.0, 56,666.0)	0.543
Ferritin Δ% (0–5)	5.3 (−85.1, 1,822.4)	−2.4 (−95.5, 2,354.2)	−16.3 (−68.8, 325.4)	−66.6 (−90.3, 2,901.4)	0.384
Ferritin D7 (ng/mL)	2,220.5 (468.0, 7,040.0)	2,511.0 (438.0, 39,912.0)	8,756.0 (491.0, 35,139.0)	2,386.0 (543.0, 84,463.0)	0.267
Ferritin Δ% (0–7)	−2.7 (−92.0, 758.0)	−37.8 (−91.6, 1,944.0)	−14.8 (−82.6, 452.8)	−13.1 (−92.8, 4373.7)	0.993
Ferritin D9 (ng/mL)	1,683.0 (638.0, 12,0659.0)	1,384.0 (181.0, 12,802.0)	3,720.0 (552.0, 35,299.0)	2,406.0 (491.4, 6682.0)	0.763
Ferritin Δ% (0–9)	−14.5 (−95.2, 525.9)	−46.5 (−97.5, 527.0)	−37.4 (−80.4, 18.8)	−29.2 (−94.0, 484.3)	0.967

Values are presented as median (Minimum, Maximum). Δ%, percentage change in ferritin.

## 4 Discussion

In our study, we found significant associations between clinical outcomes in patients with HPS and various laboratory parameters, treatment modalities, and demographic factors. The patient cohort had a nearly balanced gender distribution and a median age indicative of a middle-aged population. The association of HPS with infections, rheumatological conditions, malignancies, and other causes highlights the diverse etiology of this syndrome. The wide range of treatments, including cyclosporine, IVIG, methylprednisolone, cyclophosphamide, and plasmapheresis, reflects the complex, and multifaceted approach required to manage this condition. Significant differences in mortality were associated with specific laboratory markers, including INR, triglyceride levels, direct bilirubin levels, and platelet count. Age and LDH levels emerged as significant predictors of mortality, with higher values associated with an increased risk of death. In our multivariate Cox regression model, older age was independently associated with increased mortality, consistent with previous reports suggesting that immunosenescence and age-related comorbidities may exacerbate the course of hyperinflammatory syndromes such as HPS. These findings suggest that certain laboratory parameters may serve as prognostic indicators in HPS.

Valuable prognostic factors have been reported in the literature. Zhang et al. (16) found that older age (>60 years), low platelet count, and high LDH levels were independent predictors of early death. In the same study, 54% of patients who died had ferritin levels above 10,000 ug/L (*p* < 0.001). Similarly, many studies have shown that thrombocytopenia is associated with poor prognosis (11, 17). Increased LDH, which can reflect tissue damage (18, 19) was also reported to be a poor prognostic factor for HPS patients, consistent with our findings. Prognostic scoring systems have also been investigated. In a study involving 226 HPS patients with an etiology of hematological malignancy, the authors developed an index they called the HLH inflammatory index by combining soluble CD25 (>3,900 U/mL) and ferritin (>1,000 ng/mL) measurements to diagnose HPS and used it as a mortality predictor (20). Based on a study including 162 adult patients, Zhang et al. established a simple scoring system for long-term survival of HPS patients dividing into three risk groups. The prediction model variables were gender, activated partial thromboplastin time, LDH, and CRP. Two-year survival rates decreased with increasing risk scores (16). Yang et al. (21) determined that patients with a poor prognosis had a higher age of onset, elevated levels of bilirubin, low platelet counts, and low fibrinogens and albumin levels. However, since these parameters are not disease-specific, the use of dynamic variations of these parameters may not be appropriate.

Ferritin, which was the primary focus of the present study, has been the subject of many studies. In a study on secondary HPS involving 106 intensive care patients, multivariate analysis showed that only serum ferritin levels were significantly associated with mortality. It was determined that a serum ferritin level above 2,000 μg/L was a predictor of mortality, with a sensitivity of 71% and a specificity of 76% (22). Abou Shaar et al. identified increasing ferritin levels as predictors of early death in adult HPS patients, emphasizing the prognostic value of ferritin in this patient population. Interestingly, increasing ferritin levels during the first week of admission (data available for 44 patients) was the only significant predictor of 30-day mortality (13). Hua et al. (23) investigated the significance of serum ferritin levels in predicting the response to induction therapy and found that ferritin values 1–4 weeks post-induction were predictive of remission. In another HPS cohort, post-treatment a serum ferritin level >1,050 ng/ml (AUC 0.775, 95% CI: 0.682–0.852, sensitivity 89.3%, *p* < 0.001) was found to be an independent prognostic marker of one-year mortality. Significantly higher survival rates were observed in the group with a decrease in ferritin levels of more than 81% compared to the group with an increase of more than 14% (94% vs. 31%, *p* < 0.001) (4). Feng et al. introduced the ferritin/platelet ratio as a practical index for predicting induction response in HPS, further supporting the role of ferritin as a key biomarker in managing the disease. In this study, the response of the patients to treatment was evaluated, and weekly ferritin levels, platelet counts, and ferritin level changes (before and after treatment) were noted. A declining serum ferritin ratio predicted treatment response in weeks 1, 3, and 4, and ferritin levels and the ferritin/platelet ratio predicted treatment response in weeks 1–4 (24). A single-center observational pilot study evaluating the treatment response of HPS patients found a significant decline in serum ferritin levels on the 10th day of treatment (25). This suggests that a decreased ferritin level in survivors is a marker that should be evaluated in the first weeks. In our study, median ferritin levels were 2,842.0 ng/mL on admission, 3,458.0 ng/mL by day 3, and then decreased to 2,699.5 ng/mL by day 5, 2,464.0 ng/mL by day 7, and finally to 1,937.0 ng/mL by day 9 of treatment. When comparing ferritin levels in relation to mortality on days 3, 5, and 7 of treatment, the levels were significantly higher in patients who died compared to survivors (*p* < 0.05 for each). The multivariate analysis underscored early ferritin reduction as an independent predictor of survival, highlighting its clinical utility. The multivariate Cox regression model revealed the significance of the percentage change in ferritin levels from day 0 to day 5 (Δ% ferritin D0–D5) (*p* = 0.001). Additionally, we found that decreases below the cut-off point of 7.54% for Δ% ferritin on D0–D3 had a sensitivity of 85.19%, a specificity of 57.14%, and an AUC of 0.684 (95% CI: 0.561–0.791, *p* = 0.005) and that increases greater than the cut-off point of 36.67% for Δ% ferritin on D0–D5 had a sensitivity of 50.0%, a specificity of 85.0%, and an AUC of 0.697 (95% CI: 0.56–70.807, *p* = 0.005), respectively. Ferritin level changes offer critical prognostic information, guiding early treatment decisions in HPS. Previous research has primarily focused on the long-term prognosis of HPS and its association with ferritin levels, without a specific emphasis on the acute phase of treatment and early survival indicators. Thus, our study contributes to the scientific understanding of HPS by highlighting the dynamic nature of ferritin levels in relation to patient survival, an area that has not been extensively explored in the context of ICU survival.

Although our analysis focused on adult ICU patients, the prognostic role of ferritin dynamics in pediatric HLH has also been explored in recent studies. Oguz et al. (26) reported that while hyperferritinemia was common among pediatric patients, it was not significantly associated with mortality; instead, hypoalbuminemia (≤2 g/dL) was a strong predictor of poor outcome. Similarly, Bin et al. (27) identified hypoalbuminemia, severe neutropenia, and hyperbilirubinemia as independent risk factors for early death. Hao et al. (28) extended this work by demonstrating a significant positive correlation between high ferritin levels (≥7,138 μg/L) and markers of liver injury, as well as increased risk for multi-organ failure, in children with infection-associated HLH. These findings are partially parallel our observations in adult patients, suggesting that ferritin kinetics and liver function parameters may be relevant prognostic markers across age groups. However, differences in association strength and cut-off thresholds highlight the potential need for age-specific stratification in future comparative studies.

While our study provides significant insights into the prognostic value of ferritin levels in HPS, it is not without limitations. First, the retrospective nature of the study may have introduced biases related to data collection and patient selection, potentially affecting the generalizability of our findings. Additionally, the study's sample size, although adequate for preliminary analysis, may not have fully captured the heterogeneity of HPS presentations and responses to treatment across a broader population. Another limitation was the lack of standardized protocols for ferritin monitoring and treatment adjustments based on ferritin levels, which could lead to variability in clinical practice and interpretation of the results. Furthermore, our study focused primarily on the prognostic value of ferritin without exploring in depth the potential mechanisms underlying its fluctuations and their impact on HPS pathology. Future research should aim to address these limitations through prospective studies with larger, more diverse patient cohorts and standardized treatment protocols. Such studies could further elucidate the role of ferritin in HPS and refine its application in clinical practice, enhancing the precision and effectiveness of treatment strategies for this challenging condition.

In conclusion, this study elucidated the prognostic significance of dynamic ferritin levels in patients with HPS, offering a novel perspective on the management and treatment of this complex condition. Our findings reveal that an early decrease in ferritin levels within the first week of treatment is a robust predictor of improved survival outcomes. By integrating ferritin level monitoring into the clinical management of HPS, our study suggests a pathway toward more personalized care, potentially leading to better patient outcomes. The evidence presented in this study advocates for the adoption of ferritin monitoring as a standard practice in the treatment of HPS, emphasizing its value not only as a diagnostic tool but also as a critical component of prognostic assessment and treatment planning.

## Data Availability

The data analyzed in this study is subject to the following licenses/restrictions: the datasets used and/or analyzed during the current study are available from the corresponding author on reasonable request. Requests to access these datasets should be directed to miraybozgul@gmail.com.
